# Treatment with sorafenib and sunitinib in renal cell cancer: a Swedish register-based study

**DOI:** 10.1007/s12032-012-0331-8

**Published:** 2012-12-18

**Authors:** Anneli Ambring, Ingela Björholt, Eva Lesén, Ulrika Stierner, Anders Odén

**Affiliations:** 1 Nordic Health Economics, Medicinaregatan 8b, 413 90 Göteborg, Sweden; 2 Present Address: Center of Registers in Region Västra Götaland, Göteborg, Sweden; 3 Department of Oncology, Sahlgrenska University Hospital, 413 45 Göteborg, Sweden; 4 Department of Mathematical Sciences, Chalmers University of Technology, 412 96 Göteborg, Sweden

**Keywords:** Sorafenib, Sunitinib, Sequential, Register, Renal cell cancer

## Abstract

Sorafenib and sunitinib are used for renal cell carcinoma (RCC). The objective was to study the treatment duration and time to death in Swedish RCC patients on sorafenib or sunitinib as first-line or monotherapy or as sequential therapy. Patients with an RCC diagnosis were identified in the Swedish Cancer Register. Information on treatment with sorafenib and sunitinib was collected from the Swedish Prescribed Drug Register, and time of death from the Cause of Death Register. Outcome measures were duration of treatment and time to death on sorafenib or sunitinib as first-line or monotherapy and sequential therapy (sorafenib–sunitinib versus sunitinib–sorafenib). Poisson regression models were used to estimate hazard ratios (HR) with 95 % confidence intervals (CI). No difference was observed for sorafenib (*n* = 123 patients) versus sunitinib (*n* = 261 patients) in treatment duration (HR 1.00; CI 0.80–1.24) or risk for death (HR 1.30; CI 0.91–1.85) when used as first-line or monotherapy. The same applied for sequential therapy with sorafenib–sunitinib (*n* = 43 patients) versus sunitinib–sorafenib (*n* = 54 patients), HR 1.47 (CI 0.71–3.02) and HR 2.01 (CI 0.86–4.68), respectively. There was a difference between the two treatments in how the duration of first-line treatment influenced the duration of second-line treatment and time to death, in favor of starting with sorafenib. In conclusion, no difference was detected between sorafenib and sunitinib in the duration of treatment or time to death when used as first-line or monotherapy. The impact of the duration of first-line treatment differed between the two sequences, and the results indicated that sorafenib as first-line treatment is a favorable choice.

**Electronic supplementary material:**

The online version of this article (doi:10.1007/s12032-012-0331-8) contains supplementary material, which is available to authorized users.

## Introduction

Renal cell carcinoma (RCC) accounts for nearly 2 % of all malignancies in the adult Swedish population [1]. Close to a thousand new cases are diagnosed every year in Sweden. For metastatic RCC, the median survival was estimated to 6–12 months in 2003 [2]. RCC is resistant to conventional therapies such as chemotherapy, radiotherapy, and hormonal therapy. The only two treatment alternatives available for inoperable or metastatic disease until a few years ago were interferon alpha (IFN-α) and interleukin 2 (IL-2) therapy. Only a small proportion of patients was eligible for and responded well to these treatments. Improved understanding of the molecular mechanisms associated with the disease, including increased transcription of vascular endothelial growth factor (VEGF), platelet-derived growth factor, and epidermal growth factor, has led to the development of multiple agents targeting RCC-promoting pathways. Sorafenib and sunitinib are two multiple tyrosine kinase inhibitors (TKIs) that, among other actions, block the intracellular domain of the VEGF receptor. Inhibition of the VEGF pathway decreases vascularization and endothelial cell proliferation and obstructs tumor growth. The two drugs were approved by the US Food and Drug Administration (FDA) in 2005/2006 and the European Medicines Agency in 2006 and have ameliorated the prognosis of advanced RCC [3–6].

Although the new therapeutic opportunities have improved the treatment of patients with RCC, patients will eventually develop tumor progression due to drug resistance or toxicity leading to dose reduction and inadequate drug levels. Clinical experience has shown that when resistant to one of the TKI agents, patients can often benefit by switching to the other, resulting in sequential use of the two drugs, sorafenib and sunitinib, respectively. These experiences suggest that only limited cross-resistance exists between the two agents [7, 8]. While several new treatment options for RCC have recently become available, such as other TKIs and mammalian target of rapamycin (mTOR) inhibitors, the optimal sequence for these various drugs has not yet been established [9, 10].

The majority of the previous studies on sequential therapy comparing sorafenib followed by sunitinib to sunitinib followed by sorafenib found that the sequence by which the agents are administered affected patient outcomes, where sorafenib followed by sunitinib appeared to be the most favorable sequence [7, 11–17]. In one of these studies, the probability of proceeding to second-line treatment was higher among patients starting with sorafenib compared to those starting with sunitinib [13]. This could indicate differences in effectiveness, tolerability and/or survival rates while on first-line treatment with sorafenib versus sunitinib. However, the analyses in all of these previous studies have included only patients who were treated with sequential therapy. This creates an artificial survival advantage, since only patients surviving long enough to start with second-line treatment are included. Survivorship bias or immortal time bias is thus introduced. Immortal time refers to the time during which the outcome could not occur [18], and this should be accounted for in the analyses.

The objective of this study was to study the treatment duration and time to death in Swedish RCC patients on sorafenib or sunitinib as first-line or monotherapy or as sequential therapy.

## Materials and methods

### Data sources

This was a nationwide, observational study based on three registers covering the entire Swedish population: the Swedish Cancer Register (SCR) with information on the primary tumor and its location, cell type, and stage at the time of diagnosis for all types of cancer patients; the Swedish Prescribed Drug Register (SPDR) with information on dispensed drugs from Swedish pharmacies (i.e., not drugs provided within hospitals); and the Cause of Death Register (CDR) including information on time and cause of death. The National Board of Health and Welfare maintains all three registers and performed the register linkages using the unique personal identification number. The study was approved by the Regional Ethical Review Board in Gothenburg.

### Patient population

Patients with a diagnosis of RCC from January 1, 1980, to December 31, 2008, were first identified in the SCR. The information collected from the SCR included T-, N- and M-stages and morphological diagnosis along with date of diagnosis. The data were linked to the SPDR, and RCC patients undergoing treatment with sorafenib and/or sunitinib from 1 July, 2005, to 31 December, 2009, were included if the date of the RCC diagnosis preceded the date of the first purchase of sorafenib or sunitinib. Demographic data at the time of diagnosis were collected from the SCR. Time of death was collected from the CDR, where data were available until 8 March, 2010.

Patients treated with IFN-α (alone or in combination with bevacizumab) prior to initiation of sorafenib or sunitinib or between the two treatment regimens were excluded, that is, patients included in the study were naïve to oncologic drugs before starting with TKIs.

### Outcome measures

The outcome measures were duration of treatment and time to death on sorafenib or sunitinib as first-line or monotherapy and on sequential therapy (sorafenib–sunitinib versus sunitinib–sorafenib). Patients were assumed to be on treatment from the date of the first purchase and as long as purchases were made. The stop date was defined as the date of the last purchase plus the number of days the purchased amount would last if the recommended dose according to the Swedish physician’s desk reference [19] was used. For sorafenib, this dose was 400 mg twice daily. The dose for sunitinib was 50 mg daily for 4 weeks followed by 2 weeks off treatment, also included in the time of treatment duration.

### Statistical analyses

This study included analyses of duration of treatment and time to death among RCC patients on sorafenib or sunitinib as first-line or monotherapy and as sequential therapy. To compare the treatment groups with regard to demographics and baseline characteristics, Mann–Whitney *U* test was used for continuous variables, Fisher’s exact test for dichotomous variables, and Mantel–Haenszel chi-square test for ordered categorical variables. Estimated median and interquartile range (IQR) of the time to event was obtained from unadjusted Kaplan–Meier curves, and *p* values were calculated with log-rank tests. Poisson regression models were used to estimate hazard functions and determine hazard ratios (HR) with 95 % confidence intervals (95 % CI) and *p* values. All significance tests were two-sided at the significance level 0.05. The statistical analyses were performed using SAS version 9.2.

### Analyses of first-line or monotherapy

The outcome variable treatment duration was defined as the time from treatment start to treatment stop, death or switch to the other TKI or to IFN-α (most likely in combination with bevacizumab). Patients were censored at 31 December, 2009 (last date with information from the SPDR), if treatment was still ongoing. For the outcome variable time to death while the patient was still on treatment, censoring occurred at treatment stop, switch to the other TKI or to IFN-α (most likely in combination with bevacizumab), or on the last date with information from the SPDR (31 December, 2009).

The adjusted Poisson regression models included all baseline variables both statistically associated with the exposure category (sorafenib versus sunitinib) and with the outcome variables treatment duration and time to death, respectively. The rationale for considering calendar year for treatment start as a potential confounder was that the use of sorafenib and sunitinib was unevenly distributed over time, where sorafenib was more commonly used in the beginning and sunitinib was more commonly used in the end of the study period.

### Analyses of sequential treatment

In an initial analysis of duration of treatment and time to death on sequential treatment, only patients treated with sorafenib followed by sunitinib and with sunitinib followed by sorafenib were included, and the same statistical analyses as now applied for first-line or monotherapy were used. To avoid immortal time bias in subsequent analyses, patients surviving first-line therapy were assumed to be subjected to second-line treatment, thereby accounting for the risk of death or treatment stop among patients treated with monotherapy, that is, sorafenib or sunitinib only. Four hazard functions (h1–h4) were developed with Poisson regression models; h1–h2 concerns first-line treatment, and h3–h4 concerns second-line treatment. All patients initiated on sorafenib or sunitinib were included in the estimation of h1–h2, independently of whether or not they were subject to second-line treatment thereafter. The functions h3–h4 included only patients for whom sequential treatment (sorafenib–sunitinib and sunitinib–sorafenib) was observed.

An overview of the hazard functions is given in Table 1. Please, see the online Supplementary Material for a more detailed description. In h1 and h2, the follow-up started from the initiation of first-line or monotherapy and ended at death or stop of first-line treatment (h1) or treatment stop only (h2). In h3 and h4, the follow-up started at the initiation of second-line treatment and ended at the stop of second-line treatment (h3) or death (h4). A direct switch to second-line therapy after discontinuation of first-line therapy was assumed, that is, the time between the two treatments was not included. Time on first-line treatment was included as a covariate in h3–h4, and calendar year for treatment start was included as a covariate in all functions.

**Table 1:** Hazard functions for analyzing the duration of treatment and time to death without the assumption of patients surviving first-line treatment

Hazard function	h1	h2	h3	h4
Type of event	Death or treatment stop	Treatment stop	Treatment stop	Death
Period	First-line or monotherapy	First-line or monotherapy	Second-line therapy	Second-line therapy and the time beyond
Start	First purchase of first-line sorafenib or sunitinib	First purchase of first-line sorafenib or sunitinib	First purchase of second-line sorafenib or sunitinib	First purchase of second-line sorafenib or sunitinib
End	End of medication supply, purchase of other treatment^a^, or death, whichever occurred first	End of medication supply or purchase of other treatment^a^, whichever occurred first	End of medication supply, purchase of other treatment^a^ or death, whichever occurred first	Death
Censoring	End of data from SPDR	End of data from SPDR or death	End of data from SPDR	End of data from CDR

*CDR* cause of death register, *SPDR* Swedish prescribed drug register

^a^Other treatments for first-line sorafenib and the sequence sunitinib–sorafenib are sunitinib or interferon alpha. Other treatments for first-line sunitinib and the sequence sorafenib–sunitinib are sorafenib or interferon alpha

The method briefly presented above and in more detail in the Supplementary Material has also been used for the statistical application in FRAX [20], also developed by one of the co-authors, professor Anders Odén. The FRAX tool was created by the World Health Organization (WHO), and the algorithm calculates fracture probability based on Poisson regression models from which hazard functions are estimated.

## Results

### First-line or monotherapy

First-line or monotherapy with sorafenib was observed among 123 patients and with sunitinib among 261 patients. No differences in demographics or baseline characteristics were observed between the two groups, except for calendar year for treatment start (Table 2). In the unadjusted Poisson regression analysis, calendar year for treatment start was a statistically significant predictor for time to death (HR 0.77; 95 % CI 0.63–0.95; *p* = 0.0132), but not for treatment duration (HR 1.06; 95 % CI 0.92–1.23; *p* = 0.3848). Analyses of time to death were therefore adjusted for calendar year for treatment start.

**Table 2:** Demographics and baseline characteristics of the patient population

	Sorafenib (*n* = 123)	Sunitinib (*n* = 261)	*p* value	Sorafenib–sunitinib (*n* = 43)	Sunitinib–sorafenib (*n* = 54)	*p* value
Age at diagnosis (years)			0.5872			0.1319
Mean (SD)	62.8 (10.7)	62.4 (8.7)		61.1 (8.6)	62.9 (9.3)	
Median (range)	63.9 (2.0–85.2)	63.8 (22.2–81.8)		60.7 (39.8–79.3)	63.9 (22.2–76.8)	
Age at medication start (years)			0.5396			0.3698
Mean (SD)	65.6 (10.3)	65.2 (8.6)		63.7 (9.0)	64.6 (9.8)	
Median (range)	66.1 (19.6–88.1)	65.1 (22.4–83.6)		62.9 (45.9–84.2)	65.3 (22.4–77.9)	
Sex, *n* (%)			0.7538			0.5066
Men	88 (71.5)	181 (69.3)		30 (69.8)	42 (77.8)	
Women	35 (28.5)	80 (30.7)		13 (30.2)	12 (22.2)	
Location of tumor, *n* (%)			0.3579			1.000
ICD-7 180.0 Kidney parenchyma	93 (75.6)	184 (70.5)		33 (76.7)	42 (77.8)	
ICD-7 180.9 Kidney, NOS	30 (24.4)	77 (29.5)		10 (23.3)	12 (22.2)	
Side, *n* (%)			0.6861			0.3242
Right	47 (44.8)	119 (47.8)		17 (44.7)	31 (57.4)	
Left	58 (55.2)	130 (52.2)		21 (55.3)	23 (42.6)	
T stage of primary tumor, *n* (%)			0.3878			0.3004
T1	16 (19.0)	31 (16.1)		4 (13.8)	9 (19.1)	
T2	20 (23.8)	42 (22.3)		5 (17.2)	12 (25.5)	
T3	40 (47.6)	90 (46.6)		18 (62.1)	24 (51.1)	
T4	5 (6.0)	18 (9.3)		1 (3.4)	1 (2.1)	
Tx	3 (3.6)	11 (5.7)		1 (3.4)	1 (2.1)	
Missing	39	68		14	7	
N stage (metastasis in lymph nodes), *n* (%)			0.8016			0.5148
N0	33 (39.8)	69 (40.6)		10 (34.5)	19 (47.5)	
N1	8 (9.6)	16 (9.4)		4 (13.8)	3 (7.5)	
N2	16 (19.3)	37 (21.8)		6 (20.7)	8 (20.0)	
Nx	26 (31.3)	48 (28.2)		9 (31.0)	10 (25.0)	
Missing	40	91		14	14	
Distant metastases, *n* (%)			0.3181			1.000
M0	23 (27.7)	57 (34.3)		9 (31.0)	12 (30.8)	
M1	46 (55.4)	80 (48.2)		14 (48.3)	20 (51.3)	
Mx	14 (16.9)	29 (17.5)		6 (20.7)	7 (17.9)	
Missing	40	95		14	15	
Time from RCC diagnosis to treatment start (years)			0.5455			0.1589
Mean (SD)	2.8 (4.2)	2.8 (4.1)		2.7 (3.5)	1.7 (2.6)	
Median (range)	1.0 (0.0–21.8)	0.9 (0.0–25.4)		1.3 (0.0–13.3)	0.6 (0.1–10.9)	
Calendar year for treatment start, *n* (%)			<0.001			<0.001
2006	27 (22.0)	4 (1.5)		14 (32.6)	1 (1.9)	
2007	68 (55.3)	92 (35.2)		18 (41.9)	22 (40.7)	
2008	18 (14.6)	116 (44.4)		9 (20.9)	26 (48.1)	
2009	10 (8.1)	49 (18.8)		2 (4.7)	5 (9.3)	

*p* values for comparisons of demographics and baseline characteristics between treatment groups were calculated with Mann–Whitney *U* test for continuous variables, Fisher’s exact test for dichotomous variables, and Mantel–Haenszel chi-square test for ordered categorical variables

**Fig. 1:**
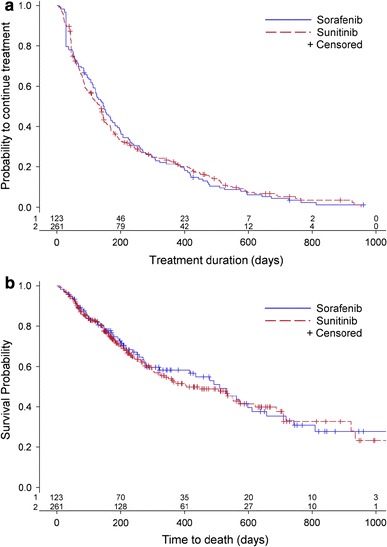
Kaplan–Meier curves of treatment duration (**a**) and time to death (**b**) with first-line or monotherapy on sorafenib and sunitinib. According to log-rank tests, *p* = 0.995 for treatment duration (**a**) and *p* = 0.767 for time to death (**b**)

The median treatment duration for first-line or monotherapy was 148 (IQR 55–299) days for sorafenib and 138 (IQR 47–296) days for sunitinib. Kaplan–Meier curves of treatment duration and time to death, respectively, are shown in Fig. 1. In the Poisson regression model, no difference was found in treatment duration between the two groups (HR 1.00; 95 % CI 0.80–1.24; *p* = 0.9696). Regarding the risk for death, no statistically significant difference was observed for sorafenib versus sunitinib; the HR in the unadjusted model was 1.04 (95 % CI 0.76–1.43; *p* = 0.7886), and 1.30 (95 % CI 0.91–1.85; *p* = 0.1468) in the model adjusted for calendar year for treatment start. The results also showed that the later the patients were initiated on treatment, the lower was the risk of death (HR for calendar year for treatment start 0.71; 95 % CI 0.57–0.90; *p* = 0.0046).

### Sequential therapy

The sequence sorafenib–sunitinib was observed among 43 patients and sunitinib–sorafenib among 54 patients. No statistically significant differences in demographics or baseline characteristics were observed between the two groups, except for calendar year for treatment start (Table 2).

According to hazard functions h1–h2, the calendar year for treatment start of first-line treatment was a statistically significant predictor for treatment stop (h2; HR 1.70; 95 % CI 1.36–2.12; *p* < 0.001), but not for the combined endpoint treatment stop or death (h1; HR 1.08; 95 % CI 0.93–1.27; *p* = 0.3216). Extended results from h1 to h2 are available in the online Supplementary Material.

**Fig. 2:**
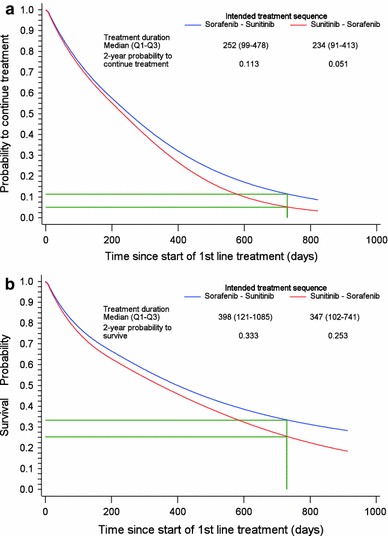
Probability to continue treatment (**a**) and to survive (**b**) with sequential therapy on sorafenib followed by sunitinib and sunitinib followed by sorafenib. The hazard functions illustrated in the figure concerns initiation of first-line treatment during year 2008. The time between the two treatments is not included. The *green lines* mark the 2-year probabilities to continue treatment (**a**) and to survive (**b**)

The median duration until treatment stop for sorafenib–sunitinib was 252 (IQR 99–478) days, and 234 (IQR 91–413) days for sunitinib–sorafenib (n.s.), when disregarding the time between first- and second-line treatments. The survival curves are shown in Fig. 2, together with the 2-year probabilities to continue treatment. No statistically significant difference was detected in the risk to stop treatment between the groups (h3; HR 1.47; 95 % CI 0.71–3.02) (see Table C, Supplementary Material for extended results). The results showed that a longer duration of first-line treatment with sunitinib was associated with an increased risk to stop second-line treatment with sorafenib (HR 2.86; 95 % CI 1.35–6.02; please, see Table C in the Supplementary Material for more details). The opposite was suggested for the time on first-line sorafenib, but this association was not statistically significant (HR 0.72; 95 % CI 0.34–1.52). The influence of the duration of first-line sorafenib was therefore compared to the duration of first-line sunitinib with a separate statistical test, described further in the Supplementary Material. The results confirmed that there was a difference in how the duration of first-line treatment with sorafenib versus with sunitinib influenced the risk to stop second-line treatment (*p* = 0.0096).

The median time until death was estimated to 398 (121–1,085) days for sorafenib–sunitinib and to 347 (102–741) days for sunitinib–sorafenib (n.s.), disregarding the time between first- and second-line treatments. Figure 2 shows the survival curves together with the 2-year probabilities to survive. The hazard function h4 is presented in Table D, Supplementary Material. The risk for death was not found to differ statistically between the treatment groups, but there was a tendency toward a higher risk in the sunitinib–sorafenib group compared to the sorafenib–sunitinib group (h4; HR 2.01; 95 % CI 0.86–4.68) (please, see Table D, Supplementary Material for extended results). The results were suggestive of a reduced risk of death with a longer duration on first-line sorafenib (HR 0.55; 95 % CI 0.21–1.43) and an increased risk with a longer duration on first-line sunitinib (HR 2.22; 95 % CI 0.97–5.09), although the associations were not statistically significant. The influence of the duration of first-line sorafenib was therefore compared to the duration of first-line sunitinib, with a corresponding statistical test as described above and further described in the Supplementary Material. The results confirmed a difference in how the duration of first-line treatment with sorafenib versus sunitinib influenced the risk of death (*p* = 0.0278).

## Discussion

No differences were detected between sorafenib and sunitinib regarding the duration of treatment or time to death when used as first-line or monotherapy or as sequential therapy. However, additional analyses showed that there was a difference between the two treatment sequences in how the duration of first-line treatment influenced the duration of second-line treatment and time to death, in favor of starting with sorafenib. There was also a trend in favor of the sorafenib–sunitinib sequence over sunitinib–sorafenib as time passed from treatment initiation. This was suggested by both the survival curves and the 2-year probabilities to continue treatment and to survive, respectively, although the 2-year probabilities were based on few observations. These findings could suggest that the outcomes for the most severely ill patients were the same regardless of treatment sequence, while a difference began to emerge among patients who survived beyond the first few months of treatment.

The results from this study are in line with several previous retrospective studies that have observed a benefit of the sequence sorafenib–sunitinib over sunitinib–sorafenib [7, 12, 15, 16]. None of the prior studies resulted in findings in favor of the sunitinib–sorafenib sequence [7, 11–17]. Sunitinib has been suggested to cause more severe adverse events compared to sorafenib [21], which could lead to a reduced vitality among patients and contribute to a shorter duration of second-line treatment. Also, sunitinib inhibits a wider range of tyrosine kinases compared to sorafenib and has a higher affinity to its targets [11, 22]. The lower affinity of sorafenib may therefore permit later salvage with sunitinib that overcomes the resistance. Further studies are needed to increase the understanding of these processes.

Sorafenib and sunitinib were made available to Swedish patients at approximately the same time, but the pattern of use showed that sorafenib was more frequently used in the early years, while sunitinib was used more commonly toward the end of the study period. The more severely ill patients tend to be treated early when new treatments are introduced, which is also supported by the higher risk of death observed among those who initiated treatment in the beginning of the study period. Also, the general increase in the use of ultrasound, computed tomography (CT) scans, and magnetic resonance imaging (MRI) over time may have led to an unintentional discovery of smaller tumors and an earlier diagnosis [9]. This could suggest that the less seriously ill patients were more commonly treated at the end of the study period. In this study, time from RCC diagnosis to initiation of first-line or monotherapy was the same in both treatment groups (2.8 years). Thus, in this regard, patients starting with sorafenib did not appear to have been unduly favored by factors relating to the timing of use.

### Strengths and limitations

To our knowledge, this is the first study investigating the outcomes of sequential treatment in cancer where no assumption was made of patients surviving first-line treatment, thereby avoiding immortal time bias. Also, this study was based on data from Swedish national health data registers covering all Swedish citizens. All patients who purchased sorafenib and/or sunitinib in a Swedish pharmacy with a diagnosis of RCC were captured. However, patients who were solely provided their drugs via participation in early access programs for RCC treatments or only received their drugs from the hospital during hospital admissions are not included in this study.

The duration from RCC diagnosis to start of TKI treatment varied between 0 and 25 years, which could indicate a heterogeneous study population. While the treatment groups were similar according to the measured variables, except for calendar year for treatment start, it cannot be excluded that the groups differed with respect to unmeasured variables. A limitation is, for example, that the data in the SCR are not updated in case of cancer progress, but derived from the first time of RCC diagnosis. Information on some factors that may influence prognosis, such as metastatic sites, performance status, or concomitant diseases, was not available. Reason for treatment discontinuation was also unavailable.

Another limitation is that actual dosage regimens were unknown. Patients were assumed to be treated according to general dose recommendations. Dosages tend to vary between patients and over time within the same patient due to adverse effects, especially toward the end of treatment. As dosages are generally lower at the end of treatment, the actual duration of treatment may have been longer than reported in this study. Also, the time between first- and second-line treatments was not included. Collectively, these factors could have contributed to the somewhat shorter treatment duration observed in this study compared to previous studies.

The data in this study suggest that the median survival time is less efficient in detecting differences between treatments in diseases with a short survival probability, as in the case of RCC. A more relevant approach would perhaps be to study survival probability at a later time point when the most critical period after treatment initiation has passed.

## Conclusion

No difference was detected between sorafenib and sunitinib in the duration of treatment or time to death when used as first-line or monotherapy. However, the impact of the duration of first-line treatment differed between the two sequences, and results indicated that sorafenib as first-line treatment is a favorable choice.

## Electronic Supplementary Material

Below is the link to the electronic Supplementary Material.

Supplementary Material 1 (DOCX 34 kb)
