# Supplementary material for: Treatment with sorafenib and sunitinib in renal cell cancer: a Swedish register-based study
Source: Med Oncol. 2012 Dec 18;30(1):331. doi: 10.1007/s12032-012-0331-8 (PMC3586400; doi:10.1007/s12032-012-0331-8)
Supplement: Supplementary file 1 — Supplementary Material 1 (DOCX 34 kb) [file 12032_2012_331_MOESM1_ESM.docx]

Online supplement to “*Treatment with sorafenib and sunitinib in renal cell cancer: a Swedish register-based study*”

Medical Oncology

Anneli Ambring, Ingela Björholt, Eva Lesén, Ulrika Stierner, Anders Odén

**Corresponding author**

Ingela Björholt

Nordic Health Economics, Medicinaregatan 8b, SE-413 90 Gothenburg, Sweden

Tel: +46 31 724 22 01

E-mail: [ingela.bjorholt@nordichealtheconomics.se](mailto:ingela.bjorholt@nordichealtheconomics.se)

**Contents**

1. Additional description of the hazard functions h1-h4
2. Extended results from the hazard functions h1-h4
3. Statistical comparison of the duration of first-line treatment between the treatment groups

**1. Additional description of the hazard functions h1-h4**

The following information is complementary to that given in the main article, and these documents should preferably be read together.

Calendar year for treatment start was shown to be the only important covariate in the initial analyses of sequential treatment, and was therefore adjusted for in the analyses *a priori*. The results of the initial analyses also showed that the event rate was considerably higher during the first six months of treatment. Consequently, two piecewise linear time variables with a break point at 0.5 years were also included in the estimation of the hazard functions: min(time in study,0.5 years) and max(time in study-0.5 years,0). These two variables were used to gain a better estimate of survival curves allowing for a different impact of time before and after 0.5 years.

The probability that the total duration of sequential treatment, *i.e.* the random variable X, exceeds a time point x, P(X>x), was calculated as follows, where z = the duration of 1^st^ line treatment:

$$P(X>x)=\int_{0}^{x} \exp(-\int_{0}^{z} h_{1}(u)du)\cdot h_{2}(z)\cdot\exp(-\int_{0}^{x-z} h_{3}(z,v)dv)dz+\exp(-\int_{0}^{x} h_{1}(u)du)$$

The event of reaching time x without death and without discontinuing second-line treatment was divided into a set of disjointed (non-overlapping) events with switches to the second-line treatment at different times z before x. The probability to survive and switch treatment before z is

$$\exp(-\int_{0}^{z} h_{1}(u)du)$$

and the probability to switch in a small interval around z with the length ∆ is ∆⋅h_2_(z). The latter term captures patients surviving until z without switching to second-line treatment, i.e. patients observed to have been treated with monotherapy only in the SPDR. The probability to survive without discontinuing second-line treatment during the period from z to x given that the switch was at z is

$$\exp(-\int_{0}^{x-z} h_{3}(z,v)dv)$$

and corresponds to the time from start of second-line treatment until x. Finally, there is a remaining possibility to reach x without an event, namely that the switch does not occur before x. The probability of this is

$$\exp(-\int_{0}^{x} h_{1}(u)du)$$

and it represents patients still on first-line or monotherapy at the end of the study period.

The probability that time to death, *i.e.* the random variable Y, exceeds a number of y, P(Y>y) was calculated as follows, where y = time to death:

$$P(Y>y)=\int_{0}^{y} \exp(-\int_{0}^{z} h_{1}(u)du)\cdot h_{2}(z)\cdot\exp(-\int_{0}^{y-z} h_{4}(z,v)dv)dz+\exp(-\int_{0}^{y} h_{1}(u)du)$$

The event of surviving until time y was divided into a set of disjointed (non-overlapping) events according to the same principle as described above.

**2. Extended results from the hazard functions h1-h4**

**Table A** Hazard function (h1) of treatment stop or death from start of first-line treatment for sorafenib and sunitinib

| **Variable** | **β-coefficient** | **Standard Error** | **Hazard Ratio (95% CI)** | **p-value** |
| --- | --- | --- | --- | --- |
| **Intercept** | -157.9 | 160.0 |  |  |
| **min(Time in Study, 0.5 years)** | -0.420 | 0.349 | 0.66 (0.33-1.30) | 0.2289 |
| **max(Time in Study-0.5 years, 0)** | -0.015 | 0.178 | 0.99 (0.70-1.40) | 0.9332 |
| **Calendar year for start of first-line treatment** | 0.079 | 0.080 | 1.08 (0.93-1.27) | 0.3216 |
| **sorafenib versus sunitinib** | -0.061 | 0.125 | 0.94 (0.74-1.20) | 0.6263 |

**Table B** Hazard function (h2) of treatment stop from start of first-line treatment for sorafenib and sunitinib

| **Variable** | **β-coefficient** | **Standard Error** | **Hazard Ratio (95% CI)** | **p-value** |
| --- | --- | --- | --- | --- |
| **Intercept** | -1066.0 | 227.6 |  |  |
| **min(Time in Study, 0.5 years)** | 1.043 | 0.515 | 2.84 (1.03-7.79) | 0.0429 |
| **max(Time in Study-0.5 years, 0)** | 0.331 | 0.212 | 1.39 (0.92-2.11) | 0.1185 |
| **Calendar year for start of second-line treatment** | 0.531 | 0.113 | 1.70 (1.36-2.12) | <.0001 |
| **sorafenib versus sunitinib** | -0.334 | 0.172 | 0.72 (0.51-1.00) | 0.0518 |

**Table C** Extended results from the hazard function h3 of risk for treatment stop

| **Variable** | **β-coefficient** | **Standard Error** | **Hazard Ratio (95% CI)** | **p-value** |
| --- | --- | --- | --- | --- |
| **Intercept** | -241.5 | 324.0 |  |  |
| **min(Time in Study, 0.5 years)** | 0.646 | 0.713 | 1.91 (0.47-7.71) | 0.3653 |
| **max(Time in Study-0.5 years, 0)** | 0.117 | 0.444 | 1.12 (0.47-2.69) | 0.7918 |
| **Time on first-line sorafenib** | -0.335 | 0.384 | 0.72 (0.34-1.52) | 0.3830 |
| **Time on first-line sunitinib** | 1.050 | 0.381 | 2.86 (1.35-6.02) | 0.0058 |
| **Calendar year for start of first-line treatment** | 0.120 | 0.161 | 1.13 (0.82-1.55) | 0.4561 |
| **sorafenib (after sunitinib) versus sunitinib (after sorafenib)** | 0.384 | 0.368 | 1.47 (0.71-3.02) | 0.2961 |

**Table D** Extended results from the hazard function h4 of risk for death

| **Variable** | **β-coefficient** | **Standard Error** | **Hazard Ratio (95% CI)** | **p-value** |
| --- | --- | --- | --- | --- |
| **Intercept** | -14.8 | 374.8 |  |  |
| **min(Time in Study, 0.5 years)** | 2.007 | 0.946 | 7.44 (1.17-47.49) | 0.0338 |
| **max(Time in Study-0.5 years, 0)** | -0.575 | 0.389 | 0.56 (0.26-1.21) | 0.1398 |
| **Time on first-line sorafenib** | -0.592 | 0.484 | 0.55 (0.21-1.43) | 0.2215 |
| **Time on first-line sunitinib** | 0.796 | 0.424 | 2.22 (0.97-5.09) | 0.0605 |
| **Calendar year for start of first-line treatment** | 0.006 | 0.187 | 1.01 (0.70-1.45) | 0.9726 |
| **sorafenib (after sunitinib) versus sunitinib (after sorafenib)** | 0.699 | 0.431 | 2.01 (0.86-4.68) | 0.1046 |

**3. Statistical comparison of the duration of first-line treatment between the treatment groups**

In the hazard function for second-line treatment, β-coefficients describing the influence of the duration of first-line treatment were included. The β-coefficients are mathematically linked to the hazard ratio (HR) and show the momentary risk to discontinue second-line treatment. This indicates that there may have been a difference in the momentary risk to discontinue second-line treatment depending on the time the patient had been on first-line treatment with the respective drugs.

The two β-coefficients reflecting the influence of the duration of first-line treatment on SO versus SU were statistically compared. The test was based on the normal distribution. The following test statistics were applied:

(β_TI1_ - β_TI2_) / √(SE(β_TI1_)^2^ + SE(β_TI2_)^2^ – 2*Cov(β_TI1_, β_TI2_))

Where

β_TI1_ = β-coefficient for time on first-line SO = -0.335

β_TI2_ = β-coefficient for time on first-line SU = 1.049

SE(β_TI1_) = Standard error for β_TI1_ = 0.384

SE(β_TI2_) = Standard error for β_TI2_ = 0.381

Cov(β_TI1_, β_TI2_) = Covariance = 0.00307

A corresponding statistical analysis was applied on time to death, using the following values:

β_TI1_ = -0.592

β_TI2_ = 0.796

SE(β_TI1_) = 0.484

SE(β_TI2_) = 0.424

Cov(β_TI1_, β_TI2_) = 0.0806
